# Preoperative Administration of Hycet Elixir Reduces Hospital Length of Stay After Pediatric Outpatient Adeno/Tonsillectomy

**DOI:** 10.31486/toj.20.0101

**Published:** 2021

**Authors:** Luis F. Salcedo, Brooke L. LeBlanc, Sarah M. Martin, Bobby D. Nossaman

**Affiliations:** ^1^ Department of Anesthesiology, Ochsner Clinic Foundation, New Orleans, LA; ^2^ The University of Queensland Faculty of Medicine, Ochsner Clinical School, New Orleans, LA

**Keywords:** *Adenoidectomy*, *analgesia*, *analgesics–opioid*, *emergence delirium*, *length of stay*, *pain–postoperative*, *pediatrics*, *preoperative care*, *tonsillectomy*

## Abstract

**Background:** Postoperative wound pain is commonly observed in the pediatric postanesthesia care unit (PACU) following tonsillectomy, adenoidectomy, and adenotonsillectomy (adeno/tonsillectomy), which contributes to increased medical care costs and delayed facility discharge. The purpose of this study was to review the benefits of preoperative administration of Hycet elixir (2.5 mg hydrocodone and 108 mg acetaminophen per 5 mL) in a pediatric population aged 1 to 9 years following adeno/tonsillectomy.

**Methods:** Patient demographics, comorbidities, surgical and anesthetic times, need for postoperative rescue therapies, and PACU recovery and length of stay times were measured in pediatric patients who received preoperative administration of Hycet elixir (0.2 mg/kg hydrocodone) for adeno/tonsillectomy in an outpatient setting compared to a control group.

**Results:** The Hycet elixir group had significant reductions in PACU and hospital lengths of stay and significant reductions in the need for postoperative rescue analgesics. No significant differences were observed in emergence times or in the incidences of unplanned hospital admission between the control and Hycet elixir groups.

**Conclusion:** These data show that the preoperative administration of Hycet elixir is well tolerated in the pediatric patient population undergoing adeno/tonsillectomy and appears to significantly reduce the need for postoperative rescue analgesics and postoperative care times. These data support the use of preoperative administration of Hycet elixir in this patient population.

## INTRODUCTION

Postoperative wound pain is commonly observed in the postanesthesia care unit (PACU) following tonsillectomy, adenoidectomy, and adenotonsillectomy (adeno/tonsillectomy).^1-3^ Postoperative pain contributes to development of emergence delirium after general anesthesia,^2-5^ with attendant risk of self-injury, requiring additional nursing staff for management and resulting in increases in medical care costs, delays in patient discharge, and decreases in patient and care provider satisfaction scores.^2-4^ Intraoperative administration of short-acting opiates, such as fentanyl, remifentanil, or sufentanil; nonsteroidal drugs; or dexmedetomidine to ameliorate the incidence of postoperative wound pain has been used with varying results.^5-9^ Additionally, postoperative administration of Hycet elixir, a commonly administered, longer-acting, hydrocodone/acetaminophen analgesic preparation, is prescribed in our pediatric PACU for control of postoperative pain. Preemptive opiate/nonopiate analgesic regimens for diverse surgical cases are currently used in pediatric surgical centers.^10-18^

The purpose of this study was to review the benefits, if any, of preoperative administration of Hycet elixir on the need for rescue therapies for postoperative wound pain, postoperative nausea and vomiting, and emergence delirium, as well as the durations of PACU and hospital lengths of stay in children undergoing adeno/tonsillectomy.

## METHODS

Demographics, comorbidities, surgical and anesthetic times, PACU and hospital length of stay times, and need for postoperative rescue therapies were measured in 77 pediatric patients aged 1 to 9 years who received preoperative oral administration of Hycet elixir (2.5 mg hydrocodone and 108 mg acetaminophen per 5 mL) in a dose of 0.2 mg/kg hydrocodone (the Hycet elixir group). The control group was a cohort of 374 pediatric patients with similar study conditions. All patients underwent an adeno/tonsillectomy procedure.

### Statistics

Categorical variables are expressed as counts and frequencies with 95% CIs, and differences between the groups were assessed using chi-square statistics. Continuous variables with skewed distributions are expressed as medians and 25% to 75% interquartile ranges (IQRs), with differences between groups assessed by the Wilcoxon rank sum test. The Hodges-Lehmann method with CI was used to estimate the median differences for all outcome times and for estimated blood loss measurements between the 2 groups.^19^ Risk differences with CI were calculated for probabilities in the need for unplanned hospital admission and the need for postoperative rescue therapies between the 2 groups.^20^ *P* values <0.005 were taken to signify statistical significance to reduce the incidence of false discovery rates.^21^

### Sample Size Determination

The incidence of the need for rescue postoperative analgesics in children aged 1 to 9 years following adeno/tonsillectomy was estimated to be 90%, with a total CI width of 15% at this institution, which is similar to other studies.^4,5^ Based on this estimated incidence, 61 completed records were needed, with an additional 20% to minimize the need for imputation strategies. Three hundred seventy-four medical records from an existing medical record database of adeno/tonsillectomy in this age group were abstracted from January 2016 to August 2016 for comparison to the Hycet group that began afterwards.^22^

The study was supported by Ochsner Clinic Foundation which had no role in the publication of the results. This study was approved by the institutional review board of Ochsner Medical Center (IRB# 2017.099).

## RESULTS

In this study of 77 consecutive pediatric patients aged 1 to 9 years, demographics and comorbidities were compared to a prior patient care dataset under similar perioperative conditions at Ochsner Medical Center (Table 1). Median age and weight were similar in both groups. A higher percentage of American Society of Anesthesiologists Physical Status (ASA PS) I patients was observed in the control group, with a higher percentage of ASA PS II patients observed in the Hycet elixir group. ASA PS III patients were similar between both groups. A lower percentage of comorbidities was observed in the Hycet elixir group (Table 1). Supplemental multimodal regimens used during these procedures are shown in Table 2. The perioperative care times, surgical conditions, and incidences of unplanned hospital admission between the 2 groups are shown in Table 3. Patients in the Hycet elixir group had clinically significant reductions in PACU length of stay and hospital length of stay compared to patients in the control group. No significant differences were observed in the incidence of unplanned hospital admission between the 2 groups (Table 3). The need for postoperative rescue therapies in the PACU is shown in Table 4. Patients in the Hycet elixir group had clinically significant reductions in the need for postoperative analgesics and the need for supportive help compared to patients in the control group (Table 4). In addition, patients in the Hycet elixir group had a clinical reduction in the need for propofol for emergence delirium; however, the risk difference CI included the value of 1. The need for antiemetics in the PACU was not clinically different between the 2 groups.

**Table 1.** Demographic Characteristics and Comorbidities in Pediatric Patients Undergoing Tonsillectomy, Adenoidectomy, or Adenotonsillectomy

Variable	Hycet Elixir Group, n=77	Control Group, n=374
Demographics		
Age, years, median [IQR]	3.0 [1.3-5.0]	4.0 [3.0-6.0]
Sex, female	36 (46.8)	180 (48.1)
Weight, kg, median [IQR]	15.1 [12.2-19.3]	17.5 [14.7-23.1]
ASA PS		
I	4 (5.2)	89 (23.8)
II	69 (89.6)	261 (69.8)
III	4 (5.2)	24 (6.4)
Comorbidities		
Tonsillar/adenoid hypertrophy	61 (79.2)	359 (96.0)
Sleep-disordered breathing	49 (63.6)	337 (90.1)

Note: Data are presented as n (%) unless otherwise indicated.

ASA PS, American Society of Anesthesiologists Physical Status; IQR, 25%-75% interquartile range.

**Table 2.** Supplemental Regimens by Treatment Group

Medication	Hycet Elixir Group, n=77	Control Group, n=374
Preoperative steroids	11 (14.3)	55 (14.7)
Preoperative midazolam	76 (98.7)	292 (78.1)
Intraoperative steroids	53 (68.8)	346 (92.5)
Intraoperative propofol	73 (94.8)	348 (93.0)
Intraoperative fentanyl	77 (100)	355 (94.9)
Intraoperative ondansetron	48 (62.3)	330 (88.2)

Note: Data are presented as n (%).

**Table 3.** Perioperative Care Times, Surgical Conditions, and Unplanned Readmissions by Treatment Group

Variable	Hycet Elixir Group, n=77	Control Group, n=374	Chi-Square, *P* Value	Hodges-Lehmann Median Difference [CI]
Preoperative time				
Preprocedural time, min	91 [78-115]	124 [90-167]	34.6, <0.0001	35 [18-52]
Anesthesia time				
Length of anesthesia, min	58 [53-67]	67 [59-77]	27.4, <0.0001	9.0 [4.0-14.0]
Emergence/out-of-room interval, min	14 [10-17]	15 [10-20]	2.5, 0.1106	1.0 [–1.0 to 4.0]
Surgical conditions				
Length of surgical operation, h	0.32 [0.25-0.43]	0.42 [0.28-0.55]	10.4, 0.0014	0.07 [0.03-0.12]
Estimated blood loss, mL	10 [2-30]	10 [10-30]	10.3, 0.0013	0 [0-7]
Postoperative times				
PACU length of stay, min	51 [29-63]	70 [46-97]	26.6, <0.0001	21 [14-29]
Hospital length of stay, h	4.1 [3.3-5.1]	5.2 [4.2-6.4]	33.4, <0.0001	1.1 [0.72-1.45]
Unplanned hospital admission, n (%)	4 (5.2)	25 (6.7)	0.2, 0.6565	–0.014 [–0.09 to 0.07]^a^

^a^Risk difference [CI] = P (yes∣Hycet group) / P (yes∣control group), [95% CI].

Notes: Data for the Hycet elixir and control groups are presented as median [25%-75% interquartile range] unless otherwise noted. *P* values <0.005 are statistically significant.

PACU, postanesthesia care unit.

**Table 4.** Postoperative Rescue Therapy Requirements by Treatment Group

Rescue Therapy	Hycet Elixir Group, n=77	Control Group, n=374	Chi-Square, *P* Value	Risk Difference [CI]^a^
Opiates	25 (32.5)	338 (90.4)	113.5, <0.0001	–0.58 [–0.74 to –0.42]
Acetaminophen	9 (11.7)	326 (87.2)	174.2, <0.0001	–0.76 [–0.88 to –0.65]
Antiemetics	1 (1.3)	4 (1.1)	0.04, 0.8498	0.003 [0.04 to –0.04]
Propofol for emergence delirium	1 (1.3)	21 (5.6)	3.3, 0.0686	–0.044 [–0.094 to 0.007]
Need for supportive help	0 (0)	21 (5.6)	7.9, 0.0049	–0.06 [–0.09 to –0.02]

^a^Risk difference [CI] = P (yes∣Hycet group) / P (yes∣control group), [95% CI].

Notes: Data for the Hycet elixir and control groups are presented as n (%). *P* values <0.005 are statistically significant.

## DISCUSSION

In this study of 77 pediatric patients undergoing common ear, nose, and throat procedures, the preoperative administration of Hycet elixir in a dose of 0.2 mg/kg hydrocodone was well tolerated and appeared to significantly shorten postoperative care times. Although a higher percentage of patients in the Hycet group received preoperative midazolam compared to the control group, the length of anesthesia time was shorter in the Hycet group. No significant differences were observed in emergence/out-of-room time intervals or in the incidence of unplanned hospital admissions between the 2 groups. Less intraoperative ondansetron and steroids were administered in the Hycet elixir group. Risk differences calculated between the 2 groups showed clinically and statistically significant risk reductions in the need for the rescue analgesics and for supportive help in the Hycet elixir group compared to the control group in the PACU.^19,20,23^ Although a clinical reduction was observed in the need for propofol for emergence delirium in the Hycet elixir group, the CI for the risk difference contained the value of 1. The need for antiemetics in the PACU was not clinically different between the 2 groups, suggesting that the preoperative administration of the multimodal analgesic is postoperatively well tolerated.

Postoperative pain commonly occurs in preschool-aged children early in the PACU.^4^ Wound pain can contribute to the development of emergence delirium,^4,6^ and although usually short-lived, emergence delirium increases the risk of injury, hence the need for additional support personnel for these patients. With the introduction of sevoflurane, the incidence of emergence delirium has increased.^4,5^ A 2004 study of intraoperative fentanyl did not ameliorate the incidences of postoperative pain and emergence delirium,^6^ suggesting that the use of short-acting opiates does not provide the necessary postoperative analgesia. The findings suggest that preoperative administration of the multimodal analgesic Hycet elixir provides postoperative benefits by decreasing the need for rescue analgesics and supportive help in the PACU. We also observed a decrease in the need for postoperative administration of propofol. The administration of the preoperative multimodal analgesic did not increase the need for antiemetics or unplanned hospital admission. These results suggest that the multimodal analgesic improves the outcomes for pediatric patients undergoing adeno/tonsillectomy.

### Strengths and Limitations

This single-center study suffers from referral bias; the approaches and methods described in this study need to be investigated in other centers. Nevertheless, this study was a consecutive patient study with robust analyses^19,20,23^ of data following common surgical procedures. One limitation of observational studies is incomplete records that can require imputation strategies to use these records for data extraction. However, as observed in this study, electronic medical records allowed for near 100% data extraction (2.5% missing data in the control group) for statistical analyses. Additionally, observations research is the preferred design for discovery of adverse events.^24^ An additional strength of this study is the use of statistical techniques to lower the incidence of false discovery rates. False discovery rates guard against incorrect declarations of statistical significance, leading to potential changes in clinical care that are attributable to chance alone.^21,25^ For example, statistical tests that use associated *P* values set for significance <0.05 have a 30% chance of false positive results with the risk of misdirected care.^21^ The false discovery rate is the complement of the positive predictive value, with the probability of the statistically significant results being one measure for supporting causality. In the present study, statistical significance and false discovery rates were set lower, resulting in a positive predictive value ranging from 93.3% to 98.2% for the preoperative multimodal analgesic in reducing the durations of PACU and hospital lengths of stay.^21^ Finally, the use of statistics to allow measures of differences in effect size were used in this study.^19,20,23^

## CONCLUSION

These data support the use of preoperative administration of Hycet elixir in a pediatric patient population. This clinical intervention could increase the value of our health care delivery by improving the quality of the postoperative experience, while decreasing health care costs.

## Notes

*This article meets the Accreditation Council for Graduate Medical Education and the American Board of Medical Specialties Maintenance of Certification competencies for Patient Care, Medical Knowledge, and Systems-Based Practice.*
